# Expanded eligibility for HIV testing increases HIV diagnoses—A cross-sectional study in seven health facilities in western Kenya

**DOI:** 10.1371/journal.pone.0225877

**Published:** 2019-12-27

**Authors:** Rachael H. Joseph, Paul Musingila, Fredrick Miruka, Stella Wanjohi, Caroline Dande, Polycarp Musee, Fillet Lugalia, Dickens Onyango, Eunice Kinywa, Gordon Okomo, Iscah Moth, Samuel Omondi, Caren Ayieko, Lucy Nganga, Emily Zielinski-Gutierrez, Hellen Muttai, Kevin M. De Cock

**Affiliations:** 1 Division of Global HIV&TB, U.S. Centers for Disease Control and Prevention, Kisumu, Kenya; 2 HIV Prevention and Community Services, Center for Health Solutions, Kisumu, Kenya; 3 HIV Testing and Counseling Services, University of California, San Francisco (FACES), Kisumu, Kenya; 4 HIV Testing and Counseling Services, Elizabeth Glaser Pediatric AIDS Foundation, Homa Bay, Kenya; 5 HIV Testing and Counseling Services, Columbia University, ICAP, Kisumu, Kenya; 6 Kisumu County Department of Health, County Government of Kisumu, Kisumu, Kenya; 7 Homa Bay County Department of Health, County Government of Homa Bay, Homa Bay, Kenya; 8 Siaya County Department of Health, County Government of Siaya, Siaya, Kenya; 9 Division of Global HIV&TB, U.S. Centers for Disease Control and Prevention, Nairobi, Kenya; Boston University School of Public Health, UNITED STATES

## Abstract

Homa Bay, Siaya, and Kisumu counties in western Kenya have the highest estimated HIV prevalence (16.3–21.0%) in the country, and struggle to meet program targets for HIV testing services (HTS). The Kenya Ministry of Health (MOH) recommends annual HIV testing for the general population. We assessed the degree to which reducing the interval for retesting to less than 12 months increased diagnosis of HIV in outpatient departments (OPD) in western Kenya. We conducted a retrospective analysis of routinely collected program data from seven high-volume (>800 monthlyOPD visits) health facilities in March–December, 2017. Data from persons ≥15 years of age seeking medical care (patients) in the OPD and non-care-seekers (non-patients) accompanying patients to the OPD were included. Outcomes were meeting MOH (routine) criteria versus criteria for a reduced retesting interval (RRI) of <12 months, and HIV test result. STATA version 14.2 was used to calculate frequencies and proportions, and to test for differences using bivariate analysis. During the 9-month period, 119,950 clients were screened for HIV testing eligibility, of whom 79% (94,766) were eligible and 97% (92,153) received a test. Among 92,153 clients tested, the median age was 28 years, 57% were female and 40% (36,728) were non-patients. Overall, 20% (18,120) of clients tested met routine eligibility criteria: 4% (3,972) had never been tested, 10% (9,316) reported a negative HIV test in the past >12 months, and 5% (4,832) met other criteria. The remaining 80% (74,033) met criteria for a RRI of < 12 months. In total 1.3% (1,185) of clients had a positive test. Although the percent yield was over 2-fold higher among those meeting routine criteria (2.4% vs. 1.0%; p<0.001), 63% (750) of all HIV infections were found among clients tested less than 12 months ago, the majority (81%) of whom reported having a negative test in the past 3–12 months. Non-patients accounted for 45% (539) of all HIV-positive persons identified. Percent yield was higher among non-patients as compared to patients (1.5% vs. 1.2%; p-value = <0.001) overall and across eligibility criteria and age categories. The majority of HIV diagnoses in the OPD occurred among clients reporting a negative HIV test in the past 12 months, clients ineligible for testing under the current MOH guidelines. Nearly half of all HIV-positive individuals identified in the OPD were non-patients. Our findings suggest that in the setting of a generalized HIV epidemic, retesting persons reporting an HIV-negative test in the past 3–12 months, and routine testing of non-patients accessing the OPD are key strategies for timely diagnosis of persons living with HIV.

## Introduction

In 2017 there were an estimated 19.6 million persons living with HIV (PLHIV) and 800,000 new HIV infections in eastern and southern Africa, accounting for more than half of the global burden of HIV, and 44% of all new HIV infections worldwide [1]. Kenya is among the high HIV-burden countries in East Africa with approximately 1.5 million PLHIV, 53,000 new HIV infections and 28,000 AIDS-related deaths in 2017 [1, 2]. In 2015, the Kenya Ministry of Health (MOH) adopted the Joint United Nations Programme on HIV/AIDS (UNAIDS) targets to, by 2020, have 90% of PLHIV know their HIV status, 90% of those diagnosed with HIV on sustained antiretroviral therapy (ART) and 90% of those on ART with sustained viral suppression [3]. In 2017, a total of 1.12 million (75%) of the estimated 1.5 million PLHIV in Kenya had been identified and were on ART [2], and 63% had suppression of the virus [1].

Estimated HIV prevalence among adults 15–49 years of age in Homa Bay (20.7%), Siaya (21.0%) and Kisumu (16.3%) counties in western Kenya is substantially higher than the national estimate (4.9%) [2]. Together these counties accounted for approximately 26% (354,000) of adult PLHIV nationally in 2017, and 20% (72,000) of those who were not on ART [2]. As ART coverage is high (≥90%) among PLHIV who know their HIV status and are enrolled in care [4] in these counties, the majority of PLHIV who are not on ART are either unaware of their HIV infection, or aware of their HIV status but not engaged in care. HIV testing strategies that enable timely diagnosis and facilitate effective linkage to care of the remaining PLHIV are, therefore, the key to achieving epidemic control in western Kenya.

HIV service delivery data reflect a trend of diminishing yield in HIV diagnoses despite increased testing. The number of HIV tests performed in public health facilities in the three counties increased from approximately 1,172,000, of which 3.6% (42,437) were positive in 2015, to 2,869,000, of which 1.0% (28,959) were positive in 2017 [5]. Provider-initiated HIV testing and counseling (PITC) in health facilities is a key strategy for the identification of PLHIV in Kenya who do not know their HIV status [6]. Kenya MOH guidelines for PITC in health facilities recommend annual HIV testing for persons in the general population [6]. More frequent retesting is recommended for specific sub-populations including pregnant women, and those at higher risk for HIV (e.g. persons with signs or symptoms of tuberculosis (TB), sex workers, men who have sex with men, people who inject drugs).

Studies evaluating a shorter (i.e. less than annual) retesting interval for routine PITC are notably absent in the literature. We hypothesized that reducing the interval for HIV testing would identify HIV-positive persons who would otherwise have been missed under prevailing guidelines for annual testing. We conducted a retrospective analysis of routinely collected program data from seven health facilities in western Kenya that expanded eligibility for HIV testing by reducing the interval for retesting to less than 12 months. Specifically, we sought to describe eligibility for HIV testing among persons accessing the OPD, and to quantify additional HIV diagnoses attributable to reducing the interval for retesting.

## Methods

### Study sites

Seven government health facilities, two each in Homa Bay and Siaya and three in Kisumu County were purposively selected to implement expanded eligibility for HIV testing. These sites were selected because they had both a high (>800) volume of monthly OPD visits and known capacity to produced timely and complete data for routine HIV program monitoring. The sites together accounted for approximately 15% of persons screened for HIV in the OPD in the three counties, having an average of 1,000–5,000 monthly OPD visits per facility. PITC at these sites is integrated into routine OPD services according to current Kenya MOH HIV testing guidelines for informed consent, confidentiality, pre- and post-test counseling, testing procedures and linkage of HIV-infected persons to treatment and support services [6]. HIV testing is routinely recommended for all persons accessing the OPD to seek health care services (i.e. “patients”). In these high HIV-burden counties HIV testing is also offered to all non-care seekers (i.e. non-patients) accompanying patients to the OPD.

### Eligibility for HIV testing

Beginning in March 2017, eligibility for HIV testing at all seven facilities was expanded by reducing the interval for retesting to less than 12 months for all persons (patients and non-patients) 15 years and above visiting the OPD. Specifically, HIV testing was offered to clients who reported a last negative HIV test in the previous 3 to 12 months, and in the previous less than 3 months if the negative test result could not be confirmed by documentation in client or clinic records. Clients were categorized as either eligible or ineligible for HIV testing. Those reporting a previous HIV-positive test, and those with a documented HIV-negative test in the previous 3 months and no disclosed risk for HIV were ineligible. HIV-positive clients who were not enrolled in clinical services for HIV were linked to care and treatment. Eligible clients met either routine MOH (“routine”) or reduced retesting interval (“RRI”) criteria for testing. Clients meeting routine criteria for HIV testing were those who reported never having been tested, those reporting a last negative HIV test 12 or more months prior, or unknown date of last test, and those reporting recent HIV exposure or signs or symptoms of TB or a sexually transmitted infection (STI). Clients meeting criteria for a reduced retesting interval (RRI) were those who reported a last negative HIV test in the previous 12 months, specifically, in the previous 3 to 12 months, or in the previous less than 3 months if the test result could not be confirmed by documentation in client or clinic records.

### Data management and analysis

Routinely collected demographic and HIV testing information, including client status as a patient or non-patient was manually recorded by HTS counselors on standardized Presidents Emergency Fund For AIDS Relief (PEPFAR) and MOH registers. De-identified data from all patient and non-patient clients 15 years of age and older who received PITC in the OPD at the seven facilities between March 30, 2017 and December 31, 2017 were included in the analysis. HTS records were abstracted from MOH testing registers and entered at site-level into an EpiInfo^TM^ database. A unique database-specific number was generated for each record. Client names and other identifiers were not included in the site-level database or in the aggregate dataset used for analysis. Data quality checks for completeness, improbable values and other errors were performed via data checks during date entry, at site-level and through exploratory analysis of the final aggregate dataset. As no unique identifiers are included in HTS registers it was not possible to account for multiple testing events for the same person. For practical reasons, however, each testing record in this analysis was counted as a single, unique individual.

Frequencies and proportions were calculated for categorical variables. Mean and median, and associated standard deviation and interquartile range, respectively, were calculated for continuous variables. Testing outcomes were described through calculation of frequencies, yield (proportion HIV-positive among those tested) and number HIV-positive per 1,000 screened for eligibility for HIV testing, overall and stratified by age category, sex, eligibility criteria, and client type (patients and non-patients). Proportions were compared using Pearson Chi-square test and rates were compared using 95% confidence interval (CI); p-values <0.05 were significant. The protocol to conduct this analysis was approved by Institutional Review Board of Kenyatta National Hospital. The protocol was reviewed according to the U.S Centers for Disease Control and Prevention (CDC) human research protection procedures and was determined to be and approved as research but CDC was not engaged.

## Results

### Overall

**Fig 1:**
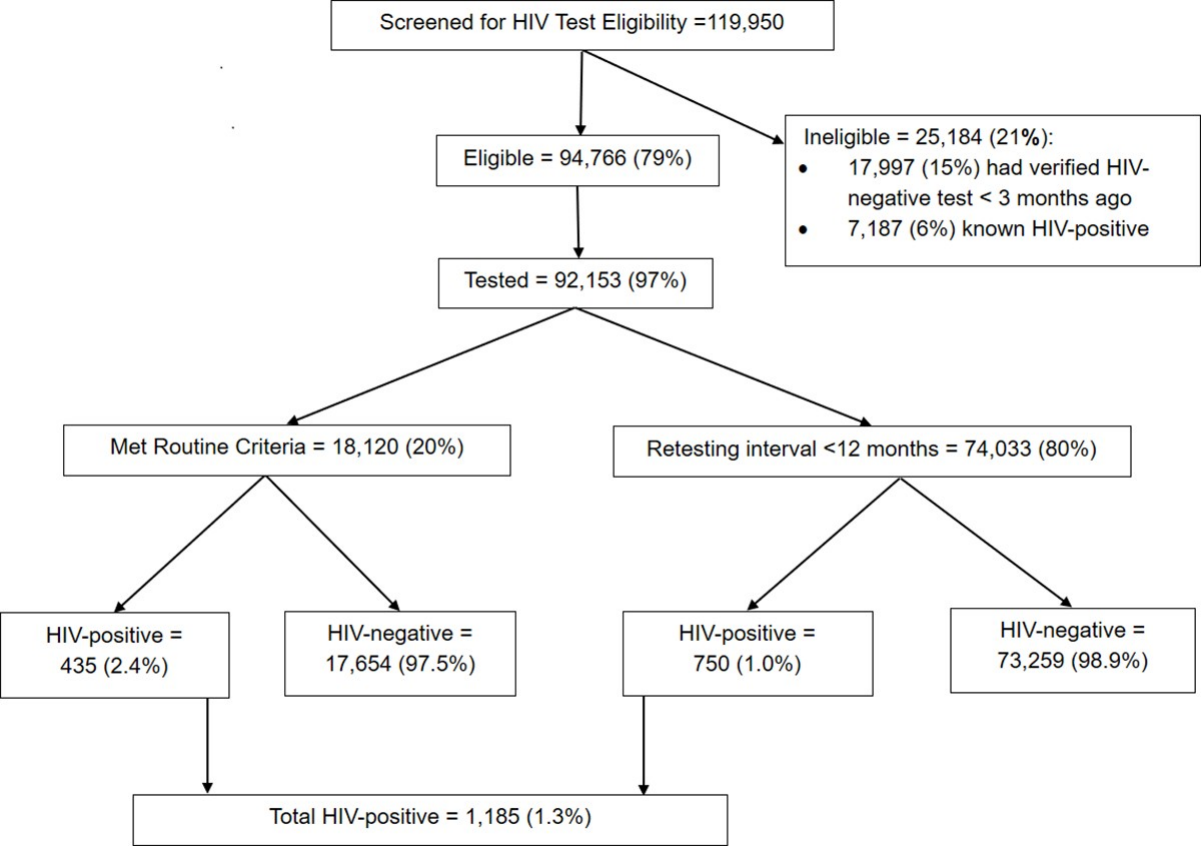
HIV Testing cascade. Of the 92,153 persons tested for HIV, 57% were female, and 60% were patients; the median age was 28 years (IQR 21–38) Table 1. A greater proportion of clients meeting RRI criteria were females (58% vs. 51%; p-value <0.001), aged 20–24 (23% vs 20%; p-value < 0.001) and 25–49 (51% vs 45%; p-value <0.001) years, and non-patients (40% vs. 38%; p-value <0.001).

A total of 119,950 persons were screened for HIV testing eligibility (Fig 1) of whom 25,184 (21%) were ineligible for testing: 6% (7,187) had known HIV-positive status, and 15% (17,997) had a verified HIV-negative test in the past three months. Of the 94,766 (79%) persons eligible for HIV testing, 97% (92,153) were tested. Of those tested, 20% (18,120) met routine criteria for testing, of whom 435 (2.4%) were HIV-positive. The remaining 80% (74,033) met RRI criteria of whom 750 (1.0%) were HIV-positive. In total 1,185 (1.3%) of all persons tested were HIV-positive.

**Table 1:** Characteristics of persons tested for HIV at selected facilities in Siaya, Kisumu and Homa Bay Counties, Kenya.

		Criteria met for HIV testing		
Characteristic	Overall N = 92,153	Routine N = 18,120	RRI N = 74,033	p-value
**Sex**	(%)	(%)	(%)	
Female	57	51	58	<0.001
Male	43	49	42	<0.001
**Age (years)**	28 (21–38)	29 (19–38)	28 (22–38)	
	(%)	(%)	(%)	
15–19	13	19	12	<0.001
20–24	22	20	23	<0.001
25–49	50	45	51	<0.001
>49	14	16	14	<0.001
**Client Type**				
Patient	60	62	60	<0.001
Non-patient	40	38	40	<0.001

The majority, 95% (87,927), of persons tested had been previously tested for HIV: 20% (18,835) had a last negative HIV test the previous 3 months, 60% (55,198) in the previous 3–12 months, 10% (9,316) 12 or more months ago, and 5% (4,578) had an unknown date of last test Table 2. The remaining 4% (3,972) had never been tested or had signs/symptoms of TB or an STI or HIV exposure (<1%).

**Table 2:** HIV diagnoses by eligibility criteria met for HIV testing among patients and non-patients tested for HIV at selected facilities in Siaya, Kisumu and Homa Bay Counties, Kenya.

Criteria met for HIV testing			Client Type			
	Overall (N = 92,153)		Patient (N = 55,424)		Non-patient (N = 36,728)	
	% of Tested	HIV+ (%)	% of Tested	HIV+ (%)	% of Tested	HIV+ (%)
**Overall**	100	1185 (1.3)	100	646 (1.2)	100	539 (1.5)
**Routine**	20	435 (2.4)	20	246 (2.2)	19	189 (2.8)
Never tested	4	110 (2.8)	4	67 (2.8)	4	43 (2.7)
HIV- test ≥12 mo ago	10	250 (2.7)	9	130 (2.5)	12	120 (2.9)
HIV- test date unknown	5	69 (1.5)	6	46 (1.3)	3	23 (2.4)
TB/STI/HIV exposure	<1	6 (2.4)	<1	3 (2.1)	<1	3 (2.8)
**RRI**	80	750 (1.0)	80	400 (0.9)	81	350 (1.2)
HIV- test 3–12 mo ago	60	611 (1.1)	56	308 (1.0)	64	303 (1.3)
HIV- test <3 mo ago	20	139 (0.7)	24	92 (0.7)	17	47 (0.8)

Overall, the yield of HIV diagnoses increased with increasing time since last reported HIV-negative test, from those tested in the past less than 3 months (0.7%), and past 3–12 months (1.1%) to those tested 12 or more months ago (2.7%) (p-value < 0.001). Clients with a retesting interval of 3–12 months accounted for 52% (611/1185) of all HIV positive persons identified, and 82% (611/750) of those with a RRI Table 2. Twenty four percent (13,082/55,198) of persons tested in the past 3–12 months had documentation of the last negative HIV test; there was no difference in yield between those with and without documentation (1.37% vs 1.32%; p-value = 0. 630).

### Differences between patients and non-patients

There were significant differences in the characteristics and outcomes of patients and non-patients. Notably, 27% (21,147/79,020) of patients versus 10% (4,037/40,929) of non-patients screened were ineligible for HIV testing: 7.8% (6,161) of patients versus 2.5% (1,026) of non-patients had known HIV-positive status, and 19% (14,986) of patients versus 7% (3,011) of non-patients had a documented HIV-negative test in the past 3 months. Uptake of HIV testing was high (>95%) in both groups. Among those tested, a greater proportion of patients were female (58% vs. 55%; p-value = <0.001), younger than 20 years (16% vs. 9%; p-value = <0.001) and older than 49 years (20% vs. 6%; p-value = <0.001) Table 3.

**Table 3:** Characteristics of patients and non-patients tested for HIV at selected facilities in Siaya, Kisumu and Homa Bay Counties, Kenya.

		Client Type		
Characteristic	Overall N = 92,153	Patient N = 55,424	Non-patient N = 36,728	p-value
**Sex**	(%)	(%)	(%)	
Female	57	58	55	<0.001
Male	43	42	45	<0.001
**Age (years)**	28 (21–38)	29 (21–45)	28 (23–35)	
	(%)	(%)	(%)	
15–19	13	16	9	<0.001
20–24	22	20	26	<0.001
25–49	50	43	60	<0.001
>49	14	20	6	<0.001

Approximately 80% of both patients and non-patients met criteria for a RRI; however, a greater proportion of patients were more recently tested having an unverified negative HIV test in the past 3 months (24% vs. 17%; p-value = <0.001). Non-patients accounted for 46% (539/1,185) of all HIV diagnoses, and 47% (350/750) HIV diagnoses among clients with a RRI. Yield was higher among non-patients overall (1.5% vs 1.2%; p-value <0.001), and across specific eligibility criteria Table 2.

### Patients

In total 646 (1.2%) of the 55,424 patients tested were HIV-positive, 62% (400) of whom met RRI criteria Table 4. Yield was similar for females and males overall (1.1 and 1.2%; p-value = 0.274, respectively) and among those meeting RRI criteria (0.9% for both sexes). In both sexes, yield increased with increasing age overall and across eligibility criteria until 49 years. Patients meeting RRI criteria accounted for greatest number of HIV diagnoses across all age bands, including: 74% (14/19) of women aged 15–19 years, 55% (38/69) of women and 76% (19/25) of men aged 20–24 years, and 66% (159/239) of women and 57% (119/208) of men 25–49 years.

**Table 4:** Number and percent of patients with an HIV-positive test result by criteria met for testing, age and sex at selected facilities in Siaya, Kisumu and Homa Bay Counties, Kenya.

Characteristic			Criteria Met for HIV Testing			
	Overall		Routine		RRI	
	Tested	HIV+ (%)	Tested	HIV+ (%)	Tested	HIV+ (%)
**Overall**	55,424	646 (1.2)	11,267	246 (2.2)	44,157	400 (0.9)
Age (years)						
**Females**	32,112	361 (1.1)	5,903	129 (2.2)	26,209	232 (0.9)
15–19	4,744	19 (0.4)	1,249	5 (0.4)	3,495	14 (0.4)
20–24	7,087	69 (1.0)	1,106	31 (2.8)	5,981	38 (0.6)
25–49	13,627	239 (1.8)	2,170	80 (3.7)	11,457	159 (1.4)
>49	6,654	34 (0.5)	1,378	13 (0.9)	5,276	21 (0.4)
**Males**	23,312	285 (1.2)	5,364	117 (2.2)	17,948	168 (0.9)
15–19	4,103	3 (0.1)	1,246	2 (0.2)	2,857	1 (0.0)
20–24	4,166	25 (0.6)	910	6 (0.7)	3,256	19 (0.6)
25–49	10,418	208 (2.0)	2,133	89 (4.2)	8,285	119 (1.4)
>49	4,625	49 (1.1)	1,075	20 (1.9)	3,550	29 (0.8)

**Fig 2:**
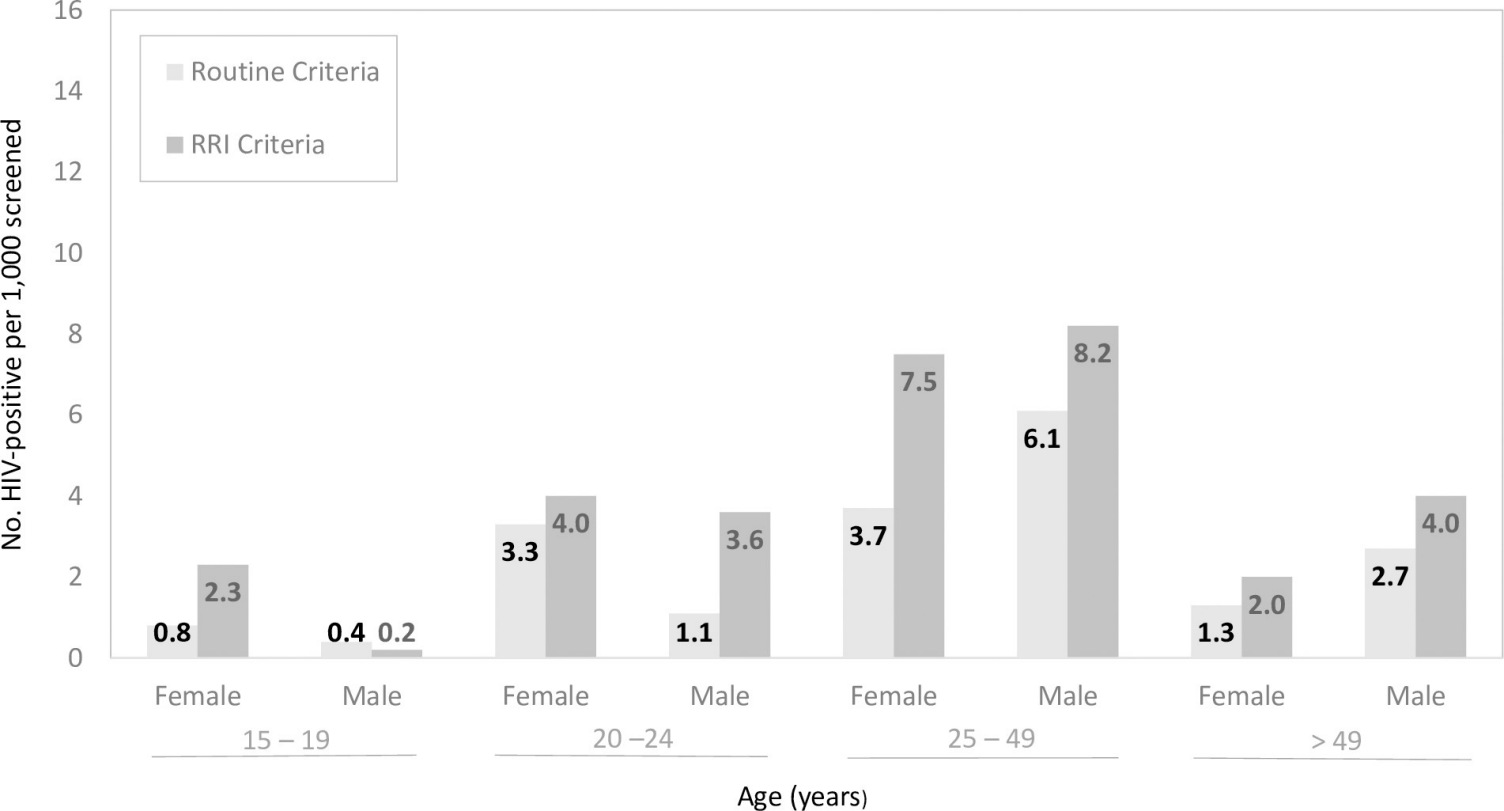
Number of HIV-positive patients identified per 1,000 screened by criteria met for testing, age and sex.

Among patients, 8.2 HIV-positive persons—3.1 meeting routine and 5.1 meeting RRI criteria—were identified for every 1,000 screened for test eligibility. For every 1,000 screened, the introduction of RRI eligibility criteria for testing resulted in the HIV diagnosis of an additional: 2.3 women 15–19 years of age, 4.0 women and 3.6 men 20–24 years of age, 7.5 women and 8.2 men 25–49 years of age, and 2.0 women and 4.0 men above 49 years of age (Fig 2).

### Non-patients

In total, 539 (1.5%) of the 36,728 non-patients tested were HIV-positive; 65% (350) of whom met RRI criteria Table 5. Yield was not statistically significantly different among females as compared to males (1.6% vs. 1.3%; p-value < .067) overall, and among those meeting RRI criteria for testing, yield was statistically significantly higher among females as compared to males (1.3% vs. 1.0%, respectively; p-value = 0.006). In both sexes, yield increased with increasing age overall and across eligibility criteria until 49 years. Non-patients meeting RRI criteria accounted for the greatest number of HIV diagnoses across all age bands, including: 68% (13/21) of women 15–19 years, 79% (76/96) of women and 65% of men (13/20) aged 20–24 years, and 67% (127/188) of women and 62% (105/169) of men aged 25–49 years.

**Table 5:** Number and percent of non-patients with an HIV-positive test result by criteria met for testing, age and sex at selected facilities in Siaya, Kisumu and Homa Bay Counties, Kenya.

Characteristic			Criteria Met for HIV Testing			
	Overall		Routine		RRI	
	Tested	HIV+ (%)	Tested	HIV+ (%)	Tested	HIV+ (%)
**Overall**	36,728	539 (1.5)	6,852	189 (2.8)	29,876	350 (1.2)
Age (years)						
**Females**	20,102	316 (1.6)	3,384	95 (2.8)	16,718	221 (1.3)
15–19	1,987	21 (1.1)	503	8 (1.6)	1,484	13 (0.9)
20–24	5,979	96 (1.6)	928	20 (2.2)	5,051	76 (1.5)
25–49	11,090	188 (1.7)	1,735	61 (3.5)	9,355	127 (1.4)
>49	1,046	11 (1.1)	218	6 (2.8)	828	5 (0.6)
**Males**	16,626	223 (1.3)	3,468	94 (2.8)	13,158	129 (1.0)
15–19	1,239	4 (0.3)	427	4 (0.9)	812	0 (0.0)
20–24	3,428	20 (0.6)	720	7 (1.0)	2,708	13 (0.5)
25–49	10,909	169 (1.5)	2,070	64 (3.1)	8,839	105 (1.2)
>49	1,050	30 (2.9)	251	19 (7.6)	799	11 (1.4)

**Fig 3:**
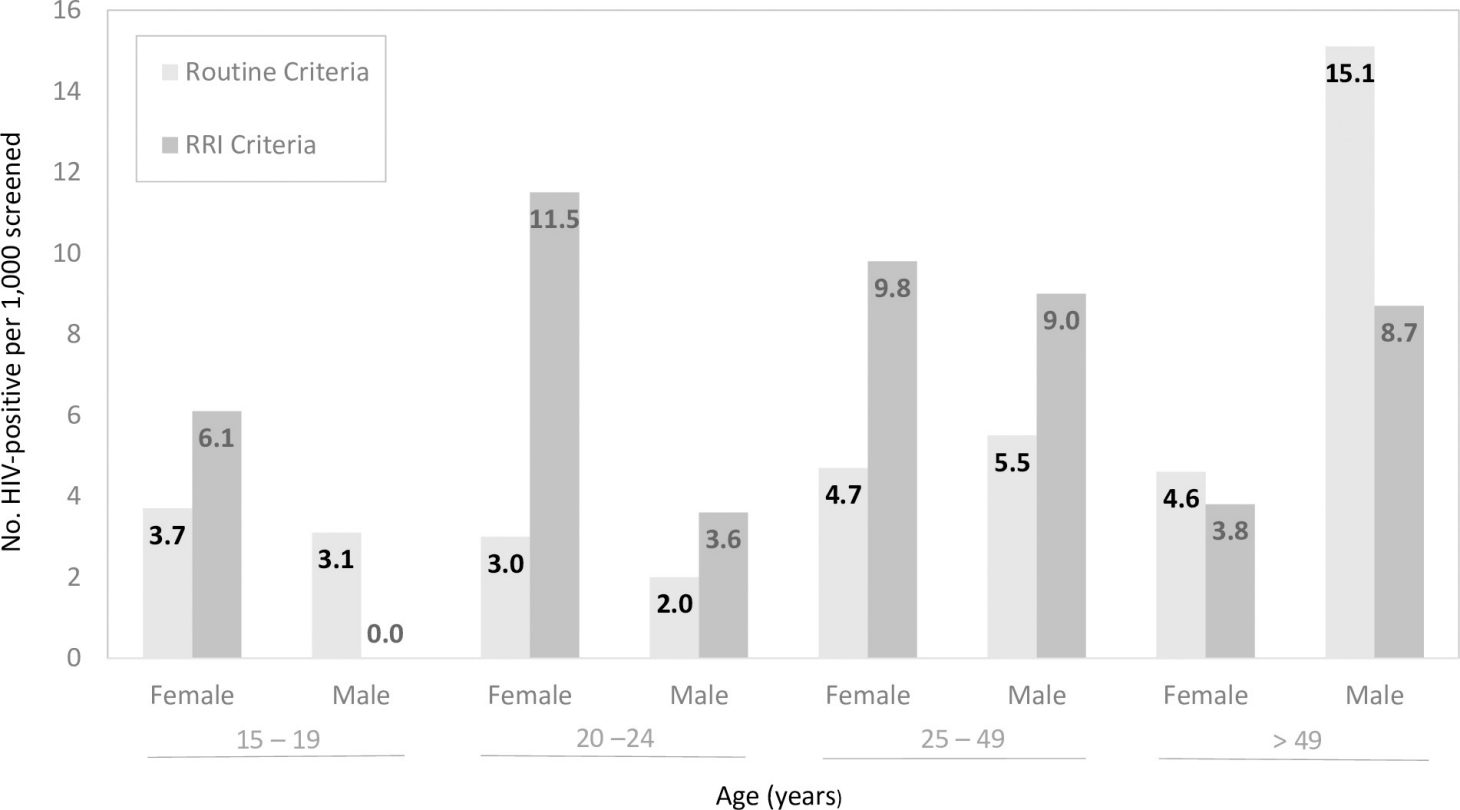
Number of HIV-positive non-patients identified per 1,000 screened by criteria met for testing, age and sex.

Among non-patients, 13.2 HIV-positive persons—4.6 meeting routine criteria and 8.6 meeting RRI criteria—were identified for every 1,000 screened for test eligibility. For every 1,000 screened, the introduction of RRI eligibility criteria resulted in the HIV diagnosis of an additional 6.1 women 15–19 years of age, 11.5 women and 3.6 men 20–24 years of age, 9.8 women and 9.0 men 25–49 years of age, and 3.8 women and 8.7 men above 49 years of age (Fig 3).

## Discussion

We demonstrated that reducing the retesting interval for HIV in the OPD can substantially increase identification of PLHIV. The majority (63%) of HIV-positive clients identified reported having a negative HIV test in the previous 12 months, and in the absence of any known disclosed risk for HIV, did not meet current routine eligibility criteria for HIV testing. We estimate that routine eligibility criteria for HIV testing would have missed approximately 5.1 HIV-infected patients and 8.6 HIV-infected non-patients for every 1,000 screened for eligibility—persons who would have remained unaware of their HIV status and infectious to others until diagnosed and initiated on treatment at a later time.

The yield (1.0%) observed among clients tested in the past 12 months in this study is higher than expected. A longitudinal survey of households in Siaya county in 2012–2016 [7] found an annual incidence of 5.7 per 1,000 person-years (i.e. 0.57%). This could be explained by misreporting of HIV status among repeat testers, and that individuals accessing the OPD might have a higher risk for HIV than the general population. Health facilities are an important point of contact with PLHIV who are unaware of their status [8]. In areas with generalized HIV epidemics, the World Health Organization (WHO) recommends universal PITC for all patients presenting to health facilities regardless of whether signs or symptoms of HIV infection are present [8]. Timely identification of PLHIV and initiation on ART reduces HIV-associated illness, averts AIDS-related deaths and prevents new HIV infections [3, 8]. A limited number of economic studies have found the cost per HIV-diagnosis made through PITC in health facilities in Kenya to vary widely from $25 [9] to $146 [10, 11]. Evaluation of the cost and cost-effectiveness of utilizing a reduced retesting interval relative to other PITC and client-initiated HIV testing strategies, would further inform establishment of an optimal testing interval for HIV programs.

Missed opportunities to diagnose HIV because of suboptimal PITC coverage have been documented in emergency departments in South Africa [12], Uganda [13] and Kenya [14], in-patient services in Uganda [15, 16], and health facilities in Ethiopia [17]. This is the first known study to demonstrate that decreasing the retesting interval for HIV to less than 12 months can further avert missed opportunities for HIV diagnosis in OPDs. With universal implementation of partner notification services across PEPFAR-supported OPDs in western Kenya beginning in 2018 [18], we expect these gains in HIV diagnosis to be further magnified through subsequent testing of index client sexual networks.

Our findings underscore the importance of extending PITC to non-patients attending OPDs. Non-patients accounted for 45% of all new HIV diagnoses, and nearly half of new HIV diagnoses among persons tested in the previous 12 months. Moreover, the yield and rate of detection of HIV-positive clients was slightly higher in non-patients than in patients, particularly in young women 15–24 years of age and women and men 25–49 years of age. Routinely collected program data does not provide information about the non-patient–patient relationship or specific risk factors for HIV infection among non-patients. Higher risk for HIV among sexual, family and social contacts of PLHIV is well documented [19]. In this study, although the testing yield was higher among non-patients, overall PLHIV were predominantly patients, with patients having a prevalence of HIV (known and newly-diagnosed) of 12.2% (6,753) as compared to 4.2% (1,560) among non-patients. The higher yield among non-patients might therefore be explained, in part, by as yet unidentified contact and/or shared risk factors with the larger sub-population of known and newly diagnosed HIV-positive patients with whom non-patients visited the OPD. Self-selection of non-patients with higher perceived risk of HIV to visit the OPD as a means to access HTS might also contribute. Evaluation of social and behavioral risk factors and the nature of relationships between patients and non-patients receiving HTS in OPDs could identify sub-populations at higher risk to better target testing and HIV prevention efforts among patient and non-patient clients in these settings.

Approximately 80% of persons accessing PITC in the OPD reported having a HIV test in the past 12 months, indicating high self-reported coverage of recent testing. We were unable to assess misreporting [17] of the date or result of the last reported HIV test in the present study; however, the relative reliability of self-reported date of last HIV-negative test is supported by both the increasing yield observed with increasing time since last reported HIV-negative test, and the observation of no difference in yield among those with and without a verified record of the last test. HIV recency testing among newly identified PLHIV who reported having a HIV-negative test in the previous 12 months would help to further assess misreporting.

This study was subject to a number of important limitations. First, findings may not be generalizable to other geographic locations and smaller health facilities. Second, it was not possible to account for multiple testing events for the same person, or to verify self-reported HIV status for persons without documentation of the last HIV test. That no difference in yield was observed among those with and without documentation of last HIV-negative test does, however, suggest a degree of reliability of self-reported status. Third, misclassification of clients as meeting RRI instead of routine criteria could have occurred (e.g. in pregnant women, HIV-negative partner in a discordant couple); however, we expect the frequency of this error to be minimized by usual client flow, including referral to antenatal and HIV care and treatment clinics, respectively, for pregnant women and discordant couples for HIV testing and other health services. Further, we did not assess self-disclosed status of men who have sex with men, sex workers or persons who inject drugs (i.e. “key populations”), as these risk factors are poorly ascertained in OPD settings. As HIV-negative persons in key populations are recommended to receive HIV testing every three months, misclassification to meeting RRI instead of routine eligibility criteria, might also have occurred. We suspect the frequency of this error to be small as county-level programmatic mapping and population size estimation conducted in 2018 found the collective size of key populations to be small (<1%) [20] relative to the general population in Homa Bay, Siaya and Kisumu Counties, and the HIV-positive yield is approximately 5% [5] among key populations tested in public health facilities in western Kenya. Finally, we did not asses linkage to care among HIV-infected persons in this analysis, which would enable a more comprehensive evaluation of the clinical cascade among newly-diagnosed PLHIV.

## Conclusions

The majority of HIV diagnoses in the OPD occurred among clients reporting a negative HIV test in the past 12 months, clients ineligible for testing under the current MOH guidelines. Furthermore, nearly half of all HIV-positive individuals identified in the OPD were non-patients. Our findings suggest that in the setting of a generalized HIV epidemic, retesting persons reporting an HIV-negative test in the past 3–12 months, and routine testing of non-patients accessing the OPD are key strategies for timely diagnosis of persons living with HIV, particularly young men and women.

## Supporting information

S1 Excel Line data for HIV testing at selected facilities in Siaya, Kisumu and Homa Bay Counties, Kenya. (ZIP)

S2 Excel Data dictionary for the line data for HIV testing at selected facilities in Siaya, Kisumu and Homa Bay Counties, Kenya. (CSV)
